# Utilization of dental care among patients with severe mental illness: a study of a National Health Insurance database

**DOI:** 10.1186/s12903-016-0280-2

**Published:** 2016-09-01

**Authors:** Po-Ren Teng, Miao-Jean Lin, Ling-Ling Yeh

**Affiliations:** 1 Department of Psychiatry, Chang Bing Show Chwan Memorial Hospital, No. 6, Lu-Gong Road, Lu-Gang Township, Changhwa County, Taiwan; 2 Department of Healthcare Administration, Asia University, No. 500, Lioufong Rd, Wufeng Taichung, 41354 Taiwan

**Keywords:** Mental illness, Dental care utilization, Oral health, Health-care surveys

## Abstract

**Background:**

The oral health of patients with severe mental illness is poor, in general, and this may be attributed, in part, to inadequate dental care. This study investigated dental care utilization among patients with severe mental illness using a national representative sample.

**Methods:**

This study used Taiwan’s National Health Insurance Research Dataset for 2009. Patients with the diagnosis of severe mental illness (ICD-9-CM: 290–298) were recruited as the study sample, and others comprised the control. Any visit to a dentist was defined as positive in terms of dental care utilization. Regression analyses were applied to determine the odds of dental care utilization for each diagnostic entity of severe mental illness, compared with the general population and controlling for potential covariates.

**Results:**

Only 40 % of 19,609 patients with severe mental illness visited the dentist within 12 months. This was significantly lower than the dental visit rate of 48.3 % for the control population (odds ratio [OR] = .72, 95 % confidence interval [CI] = .69–.74; *P* <0.0001). The odds of dental care utilization differed among the severe mental illness diagnostic categories; e.g., the odds were lowest among those with alcohol psychoses (OR = .54, CI = .43–.68), senile dementia (OR = .55, CI = .52–.59) and other organic psychoses (OR = .58, CI = .52–.65), and highest among those with mood disorder (OR = .89, CI = .85–.94), with schizophrenic patients occupying a mid-level position (OR = .63, CI = .59–.67).

**Conclusions:**

Patients with severe mental illness received less dental care than the general population. Health care providers and caregivers of patients with severe mental illness should encourage them to visit the dentist regularly, in order to improve the oral health of these vulnerable patient groups.

## Background

The significance of oral health is becoming more and more apparent. Published reports have indicated a relationship between poor oral health and many systemic diseases, including cardiovascular and respiratory diseases [1–7]. Poor oral health also has physical and psychological effects related to eating, nutrition, speech, quality of life, self-esteem and self-image. Even so, oral health is often overlooked by psychiatric personnel and neglected by the patients themselves. Poor oral health has been found among psychiatric patients in hospitals and in the community [8–16]. Studies have reported that certain factors, including amotivation, the decrease in salivary flow due to drug use, poor oral hygiene, specific dental phobias, the costs of dental visits, lack of accessible dental care facilities and infrequent dental visits, may account for this [14, 17].

Although the treatment need is huge, it has been suggested that the difficulty patients with major psychiatric diseases have in receiving dental care is due to a lack of motivation, inadequate cooperation and poor communication [18]. Many patients lack awareness of the importance of dental care [15]. They neglect making appointments for routine dental checkups and are more likely to seek dental help only when they experience pain [15]. Besides, prolonged stay in institutions may limit psychiatric patients’ ability to access dental care [19].

There have been only a few small surveys investigating dental care utilization among patients with severe mental illness [8, 12, 20, 21]. Moreover, the subjects in those studies were probably biased samples, in that they came from institutions or local outpatient clinics. Using the Danish National Patient Registry, Nielsen et al. found that only 43 % of patients with schizophrenia received dental services within 1 year [22]. To our knowledge, there are no studies that have examined simultaneously dental care utilization among patients with different diagnoses of severe mental illness, using national data.

The aims of this study were to investigate the use of dental care by patients with severe mental diseases within a 12-month period, and to compare this with dental care utilization in the general population. We hypothesized that, first, patients with severe mental diseases would receive dental services less frequently, and second, the rate of dental service utilization would differ among those disease diagnostic entities.

## Methods

### Sample

Taiwan’s National Health Insurance (NHI) program is a single-payer compulsory health insurance plan for all Taiwan residents that guarantees equal access to healthcare for all citizens. In 2003, 21,869,478 residents were enrolled in the NHI, with a coverage rate of 96 %. The Bureau of NHI (BNHI) has contracted with 17,022 medical institutions, constituting 93.8 % of medical institutions nationwide. Nearly all dental services providers are contracted with the BNHI and provide basic dental services with inexpensive co-payments. Over 90 % of dentists are private practitioners. Although patients need to make appointments with the dentists, they could register on-site if there were relatively urgent needs. By the end of 2005, approximately 22.7 million residents were enrolled in Taiwan’s NHI program, with a coverage rate of 98 %. Taiwan’s NHI program includes the National Health Insurance Research Database (NHIRD), which has collected data on the healthcare utilization of insured residents, including expenditures, medical procedures/treatments, dental services, and basic characteristics of patients, providers and physicians. Using a systematic random sampling method, the National Health Research Institute (NHRI) extracted a representative database of 1,000,000 people from the registry of all NHI enrollees to be used for research purposes; there were no statistically significant differences between this sample and all enrollees. The NHIRD uses the International Classification of Diseases, 9th revision, clinical modification diagnoses (ICD-9-CM). The data for 2009 were used for the current analysis.

All subjects in the NHIRD that met the following criteria were defined as having severe mental illness:

1. Having an inpatient diagnosis of psychoses (ICD-9-CM: 290–298) and/or at least a one-year diagnosis of same in an outpatient service. The childhood psychoses (ICD-9-CM: 299) sample was too small and was excluded from analysis.
2. Having data available for a minimum of 12 months before and after the index date.
3. Aged 18 years or older on the index date.

Data on the use of dental care was obtained from the NHIRD. Visiting any dentist within the study period for whatever reason was defined as having received dental care. For the management of covariates, age was regrouped as follows: 18–35, 35–50, 50–65 and over 65 years, respectively. The income-related insurance payment amount of our study subjects was used as a proxy measure of individual socioeconomic status (SES). SES is recognized as an important factor for dental care utilization. The study subjects were categorized into three groups: (1) low SES, lower than US$528 per month, (2) moderate SES, between US$528 and $833 per month, and (3) high SES, US$833 per month or more [23]. Subjects who received a social welfare reimbursement from the government due to lack of regular income and who self-owned their household were classified as low income. We used a dentist-to-population ratio to represent dental care accessibility. The ratio was regrouped as low (fewer than 3.42 dentists per 10,000 residents), middle (3.43 to 6.5 dentists per 10,000 residents) and high (more than 6.51 dentists per 10,000 residents), based on a lower quartile, middle half and upper quartile division. The number of outpatient visits was used as a proxy for health care utilization, and was categorized as low (fewer than 6 outpatient visits per year), middle (7–23 visits) and high use (24 visits or more). However, since patients with severe mental illness visited doctors significantly more than the control population, when exploring predictors of dental care in this group, the covariates were re-categorized as low (fewer than 18 outpatient visits per year), middle (19–48 visits) and high use (49 visits or more). In addition, hospitalization with at least 30 bed days in a year was used as a proxy for poorer health status.

### Statistical analyses

The data were processed using SPSS version 17. Logistic regression was used to determine the odds of dental visits among patients with severe mental illness and the general population. The multiple logistic regression model was further used to determine the odds of dental visits of patients in different diagnostic groups compared with the general population, controlling the following variables: gender, age group (age 18–35, 35–50, 50–65 and over 65 years), hospitalization with at least 30 bed days, socioeconomic status, dentist population density, low income status and outpatient visit frequency. The significance level was set at 0.05.

## Results

### Basic demographics

A total of 642,784 subjects were recruited for the study. Of this group, 19,609 (3.1 % of the whole group) were diagnosed as having severe mental illness. The patients with severe mental illness were significantly older than the control group (56.1 vs. 44 years; *P* < 0.001). Females were predominant among the subjects with severe mental illness (56.1 % vs. 52.8 %; *P* <0.001) (Table 1).

**Table 1:** Characteristics of patients with severe mental illness and the controls

Characteristic	Mental illness		Control		*P* value^a^
	*N*	%	*N*	%	
Gender					
Male	8606	43.9	294,036	47.2	<0.001
Female	11,003	56.1	329,139	52.8	
Age group					
18–34	3256	16.6	220,215	35.3	<0.001
35–49	5131	26.2	190,390	30.6	
50–64	4168	21.3	133,643	21.4	
65+	7054	36	78,927	12.7	
Insurance premium					
Less than US$ 528	11,631	59.3	207,414	33.3	<0.001
US$ 528–833	5830	29.7	220,778	35.4	
US$ 834 or more	2148	11	194,983	31.3	
Dentist no. per 10,000 residents					
Less than 3.43	4968	25.3	153,061	24.6	<0.001
3.43–6.50	7494	38.2	293,535	47.1	
6.61 or more	7147	36.4	176,579	28.3	
Hospitalization bed days					
Less than 30 days	17,746	90.5	618,760	99.3	<0.001
30 days or more	1863	9.5	4415	0.7	
Low income					
Yes	1196	6.1	5368	0.9	<0.001
No	18,413	93.9	617,807	99.1	
Outpatient visit no.					
0–6	889	4.5	158,353	25.4	<0.001
7–23	6083	31.1	315,280	50.6	
24 or more	12,637	64.4	149,542	24	

^a^Chi-square test

In the severe mental illness group, 7625 patients (38.9 %) had mood disorders, followed by 4772 patients (24.3 %) with senile dementia, 4023 (20.5 %) with schizophrenic disorders, 1589 (8.1 %) with other organic psychoses, 542 (2.8 %) with other nonorganic psychoses, 335 (1.7 %) with alcoholic psychoses, 305 (1.6 %) with transient organic psychoses, 275 (1.4 %) with delusional disorders and 127 with drug psychoses (0.6 %).

### Dental care utilization

Overall, only 40 % of patients with severe mental illness visited the dentist within 12 months. This is significantly lower than the dental visit rate of 48.3 % in the control population (OR, 0.72, 95 % CI, 0.69–0.74; *P* < 0.001). Of note, the dental visit rates differed significantly among the various diagnostic groups (Table 2). The dental visit rates were 27.2 % among patients with senile dementia, 32.8 % among patients with other organic psychoses, 33.4 % among patients with transient organic psychoses, 34.6 % among patients with alcoholic psychoses, 39.4 % among patients with schizophrenic disorders, 41.1 % among patients with other nonorganic psychoses, 42.5 % among patients with delusional disorders and 44.9 % among patients with drug psychoses, respectively. However, over half (50.1 %) of subjects with mood disorders visited dentists during the study period, which was a significantly higher rate than for patients with other diagnostic entities (*P* < 0.001). The rate of dental care utilization by patients with senile dementia was also lower than that of age-matched (over 60 years old) counterparts (27.2 % vs. 39.3 %).

**Table 2:** Rates and odds of dental care utilization among patients with severe mental illness

Group	Rate of dental care	No. with dental care /total No.	Adjusted odds ratio^a^ (95 % CI^b^)
General population	48.3 %	300,937/623,175	Reference
Senile dementia	27.2 %	1298/4772	0.55 (0.52–0.59)*
Alcohol psychoses	34.6 %	116/335	0.54 (0.43–0.68)*
Drug psychoses	44.9 %	57/127	0.75 (0.53–1.08)
Transient organic psychoses	33.4 %	102/305	0.60 (0.47–0.77)*
Other organic psychoses	32.8 %	521/1589	0.58 (0.52–0.65)*
Schizophrenia	39.4 %	1585/4023	0.63 (0.59–0.67)*
Mood disorders	50.1 %	3823/7625	0.89 (0.85–0.94)*
Delusional disorders	42.5 %	117/275	0.83 (0.65–1.06)
Other nonorganic psychoses	41.1 %	223/542	0.67 (0.56–0.80)*

^a^Multiple logistic regression controlling for gender, age, socioeconomic status, dentist/population ratio, hospitalization with 30 or more bed days, low income and outpatient visit numbers

^b^Confidence interval

*P < 0.001

### Multiple logistic regression

In the multiple logistic regression model, after controlling for potential confounding covariates, patients with severe mental illness had only a 70 % probability of dental care utilization compared with the control group (OR, 0.70; 95 % CI, 0.68–0.72; *P* < 0.001).

All patients with severe mental illness, except delusional disorder and drug psychoses, had a lower likelihood of visiting dentists than did the control subjects (Table 2). Although patients with mood disorders consulted dentists no less frequently than the control group in the crude analysis, they still had a lower likelihood of dental care utilization after controlling confounding factors (OR, 0.89; 95 % CI, 0.85–0.94; *P* < 0.001).

We did a logistic regression analysis limited to patients with severe mental illness only, to determine the predictors for dental care utilization. Older persons, hospitalization for more than one month, and subjects with low income were less likely to visit dentists. However, subjects residing in areas with more dentists received more dental care. Those who visited more outpatient clinics also had a higher likelihood of visiting dentists. Patients with a high SES visited dentists more frequently than those with a low SES (Table 3).

**Table 3:** Predictors of dental care utilization among patients with severe mental illness

Variable	Unadjusted odds ratio		Adjusted odds ratio	*P* value
	Estimate (95 % CI)	*P* value	Estimate (95 % CI)	
Gender				
Female	1		1	
Male	0.89 (0.84–0.94)	<0.001	0.96 (0.91–1.02)	0.207
Age group				
18–34	1		1	
35–49	0.81 (0.74–0.89)	<0.001	0.75 (0.68–0.82)	<0.001
50–64	0.86 (0.78–0.94)	0.001	0.67 (0.61–0.74)	<0.001
65+	0.49 (0.45–0.54)	<0.001	0.36 (0.33–0.40)	<0.001
Socioeconomic status				
Low	1		1	
Middle	0.97 (0.91–1.04)	0.378	0.93 (0.87–1.00)	0.053
High	1.69 (1.54–1.85)	<0.001	1.34 (1.21–1.48)	<0.001
Dentist/population ratio^a^				
Low	1		1	
Middle	1.29 (1.20–1.39)	<0.001	1.27 (1.18–1.38)	<0.001
High	1.62 (1.50–1.75)	<0.001	1.60 (1.48–1.73)	<0.001
Hospitalization bed days				
Less than 30 days	1		1	
30 days or more	0.75 (0.68–0.83)	<0.001	0.82 (0.73–0.91)	<0.001
Low income				
No	1		1	
Yes	0.85 (0.75–0.95)	0.006	0.73 (0.64–0.83)	<0.001
Health care utilization^b^				
Low	1		1	
Middle	2.08 (1.93–2.24)	<0.001	2.41 (2.23–2.61)	<0.001
High	2.82 (2.59–3.07)	<0.001	3.83 (3.50–4.19)	<0.001

^a^Low: less than 3.43, middle: 3.43–6.5, high: 6.51 or more (per 10,000 residents)

^b^umbers of outpatient visits in the year 2009; low: less than 18, middle: 19–48, high: 49 or more

## Discussion

To the best of our knowledge, this is the first report on the dental care utilization of patients with severe mental illness compared with the general population using Taiwan’s NHI database. The main results of this study indicate that, compared with the general population, patients with severe mental illness were less likely to receive dental care within 12 months. In addition, the annual rates of dental visits differed among the groups of different diagnosed psychoses.

Our results are in agreement with the literature, in that some factors, including older age [24], low income [25, 26], poorer health status [27] and lower SES [26], were associated with lower use of dental care. Furthermore, people residing in areas with few dentists had less access to dental care. Extra effort should be put forth to enhance the oral health of patients with severe mental illness and with the abovementioned factors.

Although patients with severe mental illness received more medical care services than the general population, they were less likely to receive routine dental care. Dickerson et al. reported similar findings, but they attributed this to limited dental care insurance coverage [28]. However, in Taiwan, dental care for all residents is covered by the NHI, so cost may not be the principal barrier to receiving dental care of Taiwanese patients with severe mental illness. Other factors, including the subjects’ lack of awareness of the importance of dental care, their low levels of perceived need, anxiety about visiting the dentist, difficulty in assessing the dental care needs of institutionalized patients [15], the reluctance of dentists to provide dental care, and possibly negative dental staff attitudes toward the subjects, may account for the under-utilization of the already-insured dental care services [29].

We found discrepancies in dental visiting rates among patients with different severe mental illness diagnoses. The possible causes of this are discussed below.

Teng et al. and Farnam et al. both indicated that schizophrenic patients may not have recognized the necessity of routine dental check-ups and visited the dentist only to seek help for a painful condition [15, 30]. In this regard, those patients probably suffered from prominent negative symptoms that contributed to their neglect of oral health and arranging dental visits. Moreover, even if they worried about their teeth, they may not visit a dentist due to anxiety about the treatment or a lack of trust in the dentist [31]. Also, the mental health staff in institutions may not be aware of patient dental care needs [13], and therefore offer limited access to annual dental check-ups [22]. And last, some dentists were hesitant about treating people with severe psychotic symptoms [32].

Our findings support the work of Mago and Thyvalikakath, which indicated that those who had mood disorders visited dentists less frequently [33]. Reports from individuals with mood disorders indicated that they had unmet dental care needs [34]. There are several explanations that may account for this phenomenon. First, patients with mood disorder were more likely to be irritable or uncooperative during dental visits and to have complaints related to dental care that were inconsistent with objective findings [35, 36]. Second, the patients more commonly reported a high level of dental fear that would deter them from receiving dental care earlier [37, 38]. Third, the lack of formal or informal support for patients with mood disorder would render them less likely to visit dentists when oral care is needed [33].

Although the risk of poor oral health is increased among patients with dementia, compared to their counterparts [39, 40], the likelihood of their visiting a dentist regularly is lower [41]. Dementia patients might not be able to verbalize their dental needs as a result of cognitive deficits [42]. Moreover, caregivers of persons with dementia may be hesitant to take dementia patients to visit the dentist [43]. Given that patients with dementia may be unable to express their dental discomfort, formal and informal caregivers should be educated on the benefits of regular dental check-ups in facilitating the dental health of dementia patients [41, 44].

The literature has shown that the risk of oral diseases is increased in individuals with alcohol dependence and substance use disorder [45–47]. We recruited patients with alcohol and drug psychoses whose oral health was presumably worse than that of those with substance use disorder alone. Despite the higher rates of dental problems among people with alcohol/drug use disorder, they receive less dental care [48]. Potential contributing factors to this dental care under-utilization include: the lifestyle of people with alcohol/drug use disorder is chaotic, which makes scheduling and adhering to regularly scheduled dental check-ups difficult [49]; those who use some illicit drugs (such as opiates) may feel no pain related to the dental problem, and so delay visiting the dentist; dental treatment is given a low priority due to the lack of expendable income [48]; dentists may be hesitant about providing services to those with substance use disorder [50].

Patients with severe mental illness generally had lower odds of utilizing dental services, and patients with dementia and organic mental disorder had the lowest dentist visiting rate. However, patients with mood disorder received dental care nearly as often as the control population. We postulated that the underlying cognitive function of patients may play a critical role in dentist-seeking behavior. Schizophrenia, as an example, is well-known for causing deterioration of patients’ cognitive functions, and one study reported that schizophrenic patients with worse executive cognitive functioning received less regular dental care [51]. Mood disorders, however, do not impair an individual’s cognitive functions, except in acute illness phases, and therefore the dental visit rate is close to that of the general population.

The strengths of this study include the representative national health data sample and that dental services are covered by the NHI, so cost is not the primary barrier to dental care access of patients with severe mental illness. However, there are limitations to this study that warrant consideration. First, the validity of the diagnosis of severe mental illness was uncertain, as this was derived from administrative data rather than standardized research interviews. Nevertheless, we further analyzed the data of a sub-sample of individuals who were diagnosed as having severe mental illness by psychiatric specialists, and yielded similar results. Second, information on the psychopathology and illness severity of patients was not available, so the relationship among the period of acute illness, cognitive functions, personal functionality and dental care utilization warrants further investigation. Third, some uncontrollable covariates such as educational level could confound the results, even though the proxy SES was put into consideration. Fourth, the study investigated a dataset for 1 year only; a sampling period of several years might yield more insight into the discrepancy in dental care utilization between patients with severe mental illnesses and the general population. Finally, the present analyses did not explore types of dental care, which could be preventive, restorative or emergency-based.

## Conclusions

In summary, patients with severe mental illness visit dentists much less frequently than the general population in Taiwan. Given the discrepancy in the rates of visiting dentists noticed among the different diagnoses of psychosis, the underlying reasons for under-utilization of dental services among patients in each severe mental illness category warrant further exploration. Changing patient care-seeking behavior in the short term would be difficult; therefore, healthcare authorities should organize dental care systems to facilitate meeting the dental needs of patients with psychiatric illnesses. Health care providers, including psychiatrists, dentists, nursing staffs in institutions, and caregivers should encourage patients with severe mental illness to regularly visit the dentist, so as to improve the oral health of these vulnerable patient groups.

## Abbreviations

ICD: International classification of diseases
NHI: National Health Insurance
NHIRD: National Health Insurance Research Database
NHRI: National Health Research Institute
SES: Socioeconomic status
SPSS: Statistical package for the social sciences
